# *In vivo* structured illumination ophthalmoscopy demonstration on the human retina using adaptive optics

**DOI:** 10.1364/BOE.559670

**Published:** 2025-06-24

**Authors:** Yann Lai-Tim, Laurent M. Mugnier, Léa Krafft, Antoine Chen, Cyril Petit, Pedro Mecê, Kate Grieve, Michel Paques, Serge Meimon

**Affiliations:** 1 DOTA, ONERA, Université Paris-Saclay, 92320 Châtillon, France; 2 Centre d’Investigation Clinique 1423, Quinze-Vingts National Ophthalmology Hospital, DGOS, INSERM, Paris, France; 3 At the time this research was performed, was jointly DOTA, ONERA, Université Paris Saclay and Quantel Medical, 63808 Cournon d’Auvergne, France; 4 Institut Langevin, ESPCI Paris, CNRS, PSL University, Paris, France; 5 Sorbonne Université, INSERM, CNRS, Institut de la Vision, 17 rue Moreau, F- 75012 Paris, France

## Abstract

Structured illumination microscopy (SIM) is one of the most versatile super-resolution techniques. Yet, its application to high-resolution live imaging has been mainly limited to fluorescent and stationary specimens. Here, we present advancements in SIM to jointly tackle all the challenges of imaging living samples, *i.e.*, obtaining super-resolution over an undistorted wide-field while dealing with sample motion, multiple scattering, sample-induced optical aberrations, and low signal-to-noise ratio. By using adaptive optics to compensate for optical aberrations and a reconstruction algorithm tailored for moving and thick tissue, we successfully apply SIM to *in vivo* retinal imaging and demonstrate structured illumination ophthalmoscopy with optical sectioning and resolution improvement for *in vivo* imaging of the human retina.

## 1. Introduction

In the living human body, the eye provides a unique non-invasive optical access to neurons in the retina. When observed with a sufficient numerical aperture, *in vivo* micron-scale imaging of retinal neurons can be achieved, provided that the optical defects of the eye are corrected. Adaptive optics (AO) has been used to that aim for more than 20 years [1], allowing diffraction-limited retinal imaging and thus revolutionizing the understanding of the structure and function of the normal visual system [2]. However, the optical aperture of any ophthalmoscope is eventually limited by the eye itself, via the iris. Even with chemically induced mydriasis, the diffraction limit often hinders the resolution of the smallest retinal cells. Several super-resolution imaging techniques have been developed in microscopy to go beyond the diffraction limit, but only few of them are compatible with *in vivo* retinal imaging: cslo [3], ism [4], vsd [5,6] and sim [7].

As noted by Wilson *et al.* in [8], a resolution gain can be obtained with a cslo only if the confocal pinhole diameter is smaller than one Airy disk diameter, at the expense of losing photons. It leads to a trade-off of signal-to-noise ratio (SNR) for resolution, a fundamental drawback that has led to the invention of more photon-efficient strategies, such as ism [9] or virtually structured detection (VSD) [5]. These two super-resolution techniques are similar to scanning laser microscopy where for each position of the illumination spot a wide-field image is acquired. For both methods, the correct exploitation of these wide-field images leads to a resolution enhancement. In image scanning microscopy (ISM), each detected photon is assigned to the position on the sample from which it most likely originated and this leads to a potential factor $\sqrt{2}$ gain in resolution, with no photon loss. In VSD, a digital spatial modulation of the wide-field images is exploited to reconstruct a super-resolved image with a theoretical resolution enhancement of factor 2 at most. However, VSD, ISM and confocal scanning laser ophthalmoscopy (cSLO) are all scanning techniques, prone to distortion due to eye motion during the raster scanning of the field of view. In consequence, scanning systems may resort to non-scanning wide-field images as ground truth to dewarp their images [10]. Therefore, there exists an unmet need for a super-resolved retinal imager that would be both photon-efficient and distortion-free.

**Fig. 1.**
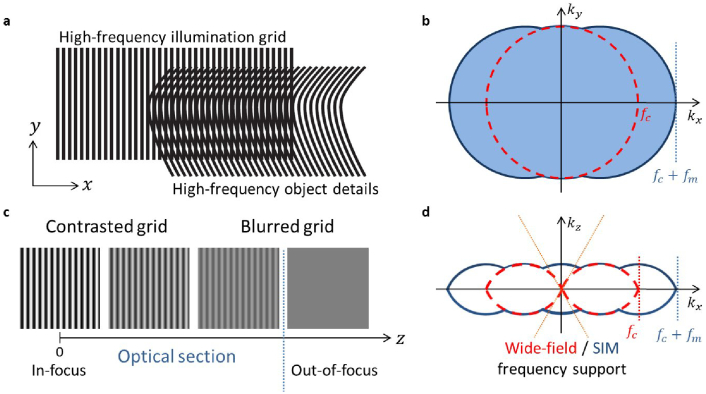
SIM principle. (a) A high-frequency sinusoidal illumination pattern is projected onto the observed object. Due to the Moiré effect (seen here as the apparent horizontal coarser lines in the overlap area), this projection down-modulates high-frequency object details that are optically unseen into the optical accessible bandwidth. (b) This enables the extraction of high-frequency details beyond the classical diffraction limit of optical imaging, which can be seen in Fourier space, as an extended frequency support (blue area) compared with the conventional wide-field bandwidth (red circle) limited by the optical cutoff frequency $f_{c}$ . It should be noted that SIM increases the conventional wide-field bandwidth by the value of the grid frequency $f_{m}$ along its modulation direction. Several orientations of the grid pattern should be used for isotropic super-resolution. (c) Additionally, as the contrast of the illumination pattern in the image focal plane decreases with defocus *z*, it is possible to discriminate the in-focus object content where the illumination grid is high contrast from the out-of-focus background where the grid is not visible due to blurring. Thus, the out-of-focus background can be computationally removed to reconstruct an optical section of the in-focus content. (d) In the reciprocal space, this optical sectioning capability can be seen in the extended SIM frequency support, which covers spatial frequencies along the $k_{z}$ axis that are missing in the conventional wide-field frequency support (yellow cone).

An alternative to ISM, VSD and cSLO that achieves both super-resolution and optical sectioning without distortion is structured illumination microscopy (SIM) [11,12]. This technique consists in illuminating the observed sample with fringe patterns. By acquiring images for different orientations and positions of the illumination patterns, a super-resolved and optically sectioned image can be reconstructed. Figure 1 illustrates the principle of the resolution enhancement and optical sectioning achieved by SIM. Recent work [7,13,14] has aimed at applying SIM to human retinal imaging but no experimental validation was provided. We identify three main issues, discussed in the above-mentioned works, which should be tackled in order to successfully apply SIM to *in vivo* retinal imaging. Firstly, eye-induced optical aberrations hinder the projection of contrasted high spatial frequency illumination patterns onto the retina and reduce the diffraction-limited passband of the instrument [15]. Secondly, the retinal motion stemming from uncontrolled eye movements [16] must be taken into account properly to avoid artefacts in reconstructions [17]. Lastly, the retina is a thick and scattering tissue. Consequently, wide-field retinal images have poor SNR due to a strong scattering background. In the case of *in vivo* imaging of mouse brain with submicron residual motion, some of these issues have been addressed in recent work by Turcotte *et al.* [18]. In the case of *in vivo* imaging of the human retina, Schock *et al.* [19] has presented a first application of structured illumination to fundus autofluorescence images, which compares fairly well with SLO system. However, as the ocular aberrations are not compensated for, the overall resolution remains lower than that of the AO imaging system.

In this paper, we describe an implementation of SIM applied to AO-corrected *in vivo* retinal imaging that addresses all of these three issues, enabling wide-field retinal imaging with optical sectioning and resolution improvement compared to conventional flood-illumination ophthalmoscopy. We have developed a structured illumination ophthalmoscope (SIO), combining a custom-made AO system [15] and a digital micromirror device (DMD) based illumination to project high spatial frequency fringe patterns onto the retina while mitigating the effects of ocular aberrations as described in the Methods. Our reconstruction technique takes the object motion and the scattering background into account so as to achieve both resolution improvement and optical sectioning on the living retina. Moreover, we propose a SIM acquisition strategy that turns the sample motion from an inconvenience to an asset.

## 2. Methods

### 2.1. Imaging system

**Fig. 2.**
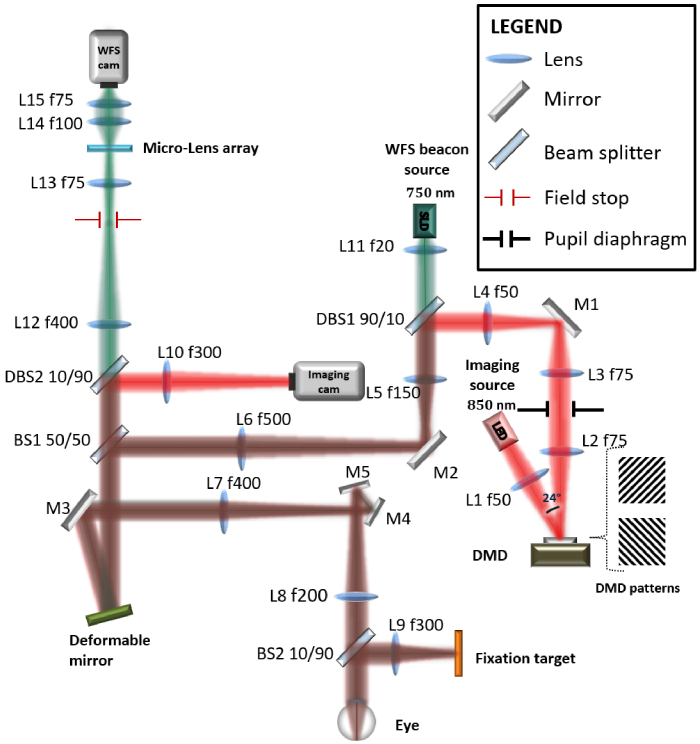
SIO schematic. All optical components are labelled: SLD, superluminescent diode; LED, light-emitting diode; DMD, digital micromirror device; L1-L15, lenses; M1-M5, plano-mirrors; BS1-BS2, beamsplitters; DBS1-DBS2, dichroic beamsplitters. The beams illustrated in red, green and brown depict respectively the illumination and detection path, the wavefront sensing path and the common (red + green) path.

To achieve super-resolution SIM imaging, coherent fringe projection techniques are usually preferred as they yield higher modulation contrast than incoherent projection techniques. However, it was shown that such projection techniques led to high speckle noise when applied to *in vivo* retinal imaging [13]. Thus, we developed an AO-corrected structured illumination ophthalmoscope (SIO) using an incoherent projection technique because it produces speckle-free SIM retinal images and it enables better optical sectioning than coherent illumination [20]. The layout of the SIO is shown in Fig. 2. It is composed of two optical subsystems: the wavefront sensing and control (WFS) subsystem and the imaging subsystem.

**Fig. 3.**
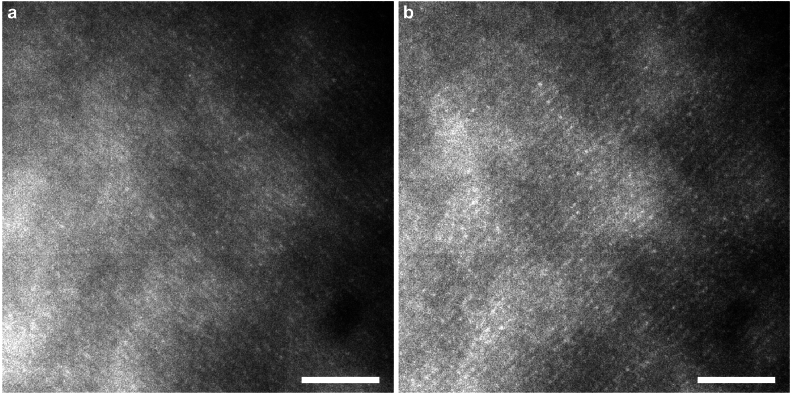
$1.24^{\circ }\times 1.24^{\circ }$ cropped individual AO-corrected SIO raw frames acquired on subject A. (a) SIO frame with a $-45^{\circ }$ oriented fringe pattern, (b) SIO frame with a $+45^{\circ }$ oriented fringe pattern. Scale bars, 0.25^∘ ^. The fringe spatial frequency corresponds to 34 cycles/degree. The cropped images are centered at 1.2^∘^ eccentricity. A linear LUT with a saturation of the 0.3% of the brightest and darkest pixels was applied to the images.

**Fig. 4.**
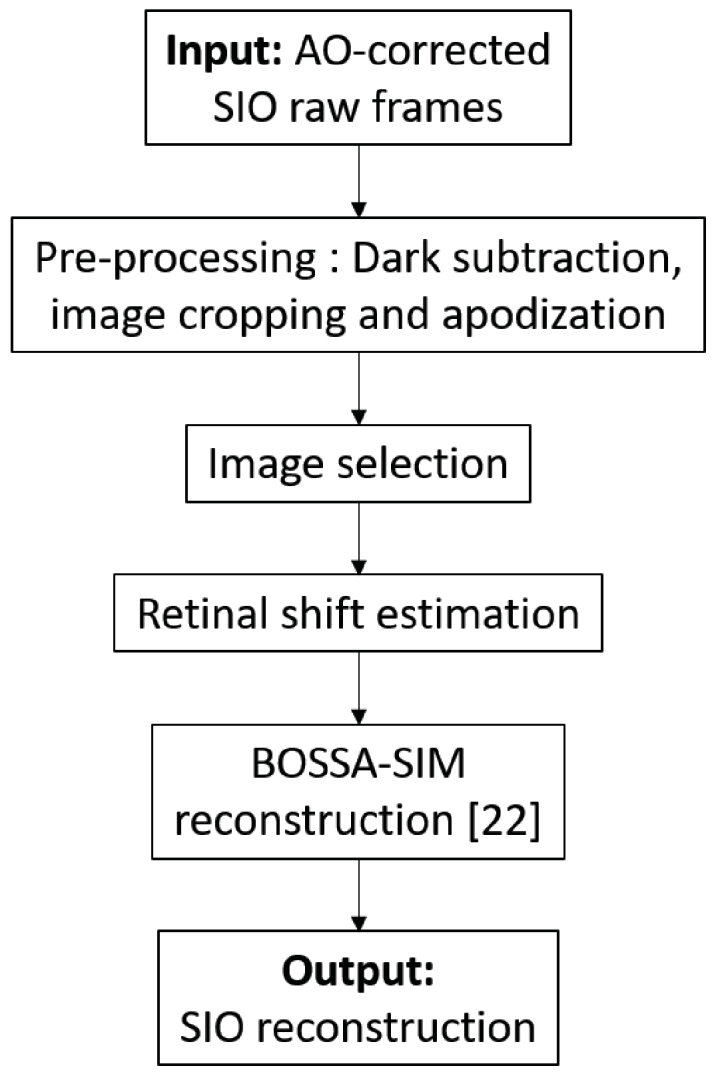
Flow diagram of the SIO reconstruction process.

The WFS subsystem enables AO correction of the illumination and detection beams and includes a fibered super luminescent diode (SLD) (LEDMOD, Omicron, Germany) centered at 750 nm, a custom-built Hartmann-Shack sensor and a deformable mirror with 97 actuators (DM97-15, ALPAO, France). More details about the AO implementation can be found here [15].

The imaging subsystem consists of a DMD-based illumination path that projects fringe patterns onto the retina using an incoherent LED source with a central wavelength of 850 nm and a spectral bandwidth of full width at half maximum 30 nm (M850LP1, Thorlabs, USA) and a detection path that directs the light backscattered by the retina toward an imaging camera (ORCA flash4-V2, Hamamatsu, Japan). In order to produce the illumination patterns, a DMD (DLP650LNIR 0.65 NIR, Texas Instrument, USA) is used as an amplitude-only spatial light modulator. It consists of an array of 1280×
800 micromirrors that can be each tilted along their diagonal to two angle positions: $+12^{\circ }$ tilt reflects the incident beam to the optical axis and $-12^{\circ }$ tilt deflects it away from the optical axis. Since the DMD micromirrors tilt along their diagonal, the DMD chip was rotated $45^{\circ }$ along the optical axis in order to keep both incident and reflected beams in one plane, parallel to the optical table. The DMD was controlled using a Vialux V-Module (V-650L, Vialux, Germany), which allows one to upload to the DMD a pattern sequence to be projected. The DMD, illuminated by a collimated LED source, was set to reflect a binary fringe patterns of adjustable spatial frequency oriented at $\pm 45^{\circ }$ toward the optical path.

These illumination patterns are then demagnified and projected onto the retina through multiple relay optics (lenses L2+L3; L4+L5; L6+L7; L8 + eye lens), fold mirrors (M1-M5) and a deformable mirror, which accounts for ocular aberrations that affect the illumination beam on its way into the eye. Even though only binary fringe patterns are produced by the DMD, a sinusoidal intensity distribution is obtained in the retina due to the limited optical bandwidth that cuts off the higher order spatial frequencies (harmonics) of the binary patterns.

Instead of accurately phase shifting the illumination patterns over the observed object as is usually done to obtain the phase diversity required for SIM reconstruction [11,12], the uncontrolled eye movements [16] introduce inter-frame retinal shifts that can be exploited to provide this phase diversity [14,21]. The SIO raw images are thus acquired using a static fringe pattern, sequentially oriented at $+45^{\circ }$ and $-45^{\circ }$ to enable two-dimensional resolution enhancement.

The imaging camera, conjugate with the DMD, collects the light backscattered by the retina enabling up to $5^{\circ }\times 5^{\circ }$ frame acquisition at 100 Hz. The backscattered beam is corrected for the ocular aberations by the deformable mirror to mitigate their effect on the acquired images. As the projected illumination pattern is focused on the retinal plane conjugate to the DMD, we can image various retinal layers by adding a defocus aberration with the deformable mirror on both the illumination and detection paths using the AO system.

### 2.2. Participants

In-vivo retinal images were captured from three healthy young participants (A: female, 25 years old; B: male, 38 years old; C: man, 23 years old). All procedures adhered to institutional guidelines and the tenets of the Declaration of Helsinki. After being informed about the study’s purpose and potential outcomes, participants provided written informed consent. The study was authorized by the appropriate ethics review boards (CPP and ANSM (IDRCB number: 2019-A00942-55)).

### 2.3. Imaging protocol

During image acquisition, each participants were seated at the SIO with head stabilization via standard chin and forehead rests, fixating on a yellow crosshair used solely to guide gaze and facilitate retinal imaging. Imaging sessions were conducted under standard conditions with neither pupil dilation nor cycloplegia, in a dark room. The pupil size was monitored during the image acquisition on the WFS camera. The pupil diameter in the eye pupil plane was evaluated to 6.7 mm for subject A, subject B and subject C with an uncertainty of about 0.18 mm on the pupil measurement. This leads to a theoretical diffraction-limited cutoff frequency $f_{c,\text{diff}}=D/\lambda$ of 140 cycles/degree, with *D* the pupil diameter at the eye and *λ* the imaging wavelength.

The onboard memory of the DMD was preloaded with our illumination patterns using the EasyProj application provided by VIALUX. The value of the fringes’ period directly influences the resolution improvement and the optical sectioning capability. A modulation frequency equal to half of the optical cutoff frequency is optimal for optical sectioning [20]. Initially, we had considered to project fringe patterns of period 2 DMD pixels (one bright and one dark, *i.e.* the smallest achievable period with our setup), corresponding to a spatial frequency of 68 cycles per degree (cpd) in the retina, which is close to the optimal modulation frequency for optical sectioning for an eye pupil diameter of about 6.7 mm. As the contrast of the 68 cpd pattern projected onto the retina was too low, we chose to increase the fringe pattern by a factor 2 to obtain exploitable fringe patterns projected onto retina. Thus, the DMD was set to project fringes of 34 cpd spatial frequency in the retina, alternatively oriented at $+45^{\circ }$ and $-45^{\circ }$ with a switching rate of 2 Hz. We set the imaging camera to acquire 2048×
2048 pixel 16-bit images with a frame rate of 100 Hz and an exposure time of 10 ms using the HCImage software (Hamamatsu, Japan). Since the switching rate of the illumination patterns is set to 2 Hz, the orientation of the fringe patterns alternates every 50 frames. Examples of acquired SIO frames are shown in Fig. 3.

When the fringe pattern is displayed, the optical power from the illumination source measured at the entrance of the eye is 850μ
W . The optical power from the WFS source at the eye’s entrance was set to 1.8μ
W . Illumination levels remained below the maximum permissible radiant exposure defined by ISO 15004-2:2007 for group 1 devices.

For each participant, a 1000-frame image sequence of total duration 10 seconds was acquired. After the image acquisition, the raw SIO frames had to be post-processed using the BOSSA-SIM reconstruction [22] to produce high-resolution images. As the BOSSA-SIM, is based on the Bayesian framework, the resolution of the reconstructed images depends on the noise level in the raw frame due to the regularization process. Consequently, increasing the number of frames to be processed improves the lateral resolution in the reconstructed image, up to the theoretical limit of SIM. It should be emphasized that 1000 raw SIO images are more than sufficient to perform a SIM reconstruction. Theoretically, 7 images per pattern orientation are enough to get the required phase diversity for SIM reconstruction according to Gruppetta *et al.* [7] assuming no noise. Here, to counterbalance the low contrast of the projected pattern and to improve lateral resolution in the reconstruction, a sequence of at least 50 images per orientation of the illumination patterns was processed for each subject. The selection of the frames to be processed and the SIO reconstruction steps are detailed in the next subsection.

### 2.4. SIO reconstruction

The different steps of the SIO reconstruction process are summarized in Fig. 4. Firstly, the 1000 SIO raw frames were pre-processed as follows. A dark frame, averaged over 1000 dark individual images, is subtracted from the raw SIO images in order to suppress the camera offset. Then the images are cropped to 1024×
1024 pixels. This image cropping is performed because we noticed that the modulation contrast of the projected pattern currently decreases in the field of view. The cropped area corresponds to the region where the fringes’ contrast is high enough to ensure reliable SIO reconstruction. The cropped images are then apodized to avoid ringing artefacts at the border of the images when performing image registration and reconstruction. The apodization is performed by multiplying the cropped images by a cosine-tapered window as, *e.g.*, in [23].

As the image quality of AO-corrected retinal images varies over time, especially preceding and during blinks, as well as during rapid and large fixational eye movements such as micro-saccades [24,25], a selection of images excluding those of poor quality is performed after the pre-processing stage. This image selection involves a three-step approach. Firstly, we retain the top 50% of the frames with the highest image quality metric. Here, the quality metric that we use is the energy of each image’s gradient, which is very similar as the image variance criterion used in [25] as both metrics measure the energy of the high frequency content in the image. Blurring smoothes out edges in the image and reduces pixel intensity variations, thus it is expected that the higher the energy of the gradient, the sharper the image. Noisier image can also imply a higher energy of the gradient but in our case, the image noise is nearly constant throughout a sequence so the chosen metric is not biased by noise. This first step efficiently removes the frames where microsaccades or blinks occur or where the quality of the AO correction is lower. Secondly we assess, for each frame, the contrast of the fringe patterns by extracting the energy at the spatial frequency corresponding to the modulation frequency of the fringes from the Fast Fourier Transform (FFT) of the frame. Among the remaining frames, we select by visual inspection the frames within an acquisition timespan of 2 seconds where the modulation contrast was high. Thirdly, in order to have an equal number of frames per illumination pattern for the BOSSA-SIM reconstruction, we discard excess frames from the most represented illumination pattern among the selected images, ensuring a balanced frame count across all patterns.

After the image selection stage, the retinal shifts are estimated in two steps: copies of the SIO selected images are first filtered to cut off the spatial frequencies of the illumination patterns so as to obtain approximate wide-field images (without the fringe pattern), then they are used as inputs in a subpixel shift estimation method designed for flood-illumination retinal images [26]. The filtering of the spatial frequencies of the patterns is performed by applying a mask equal to 0 near the modulation frequency and 1 elsewhere in the Fourier domain.

Lastly, the selected SIO pre-processed images and the estimated retinal shifts are used as inputs in the BOSSA-SIM algorithm [22] in order to obtain the reconstructed SIO image. The BOSSA-SIM algorithm allows unsupervised SIM super-resolved reconstruction for thick and moving objects. It consists of minimizing the following *Maximum a Posteriori* criterion, under positivity constraint:

(1) J(o0,od)=12∑
j=1N‖ij−
Mj(o0,od)σ
j‖22+λ
2[∑
f|o~
0(f)|2So0(f)+∑
f|o~
d(f)|2Sod(f)]

where:

•
$o=(o_{0},o_{d})$ is the 2-layer object to be reconstructed. It is composed of the in-focus object layer $o_{0}$ and the defocused object layer $o_{d}$ into which the out-of-focus contributions and scattered light are rejected so as to obtain a super-resolved and optically sectioned in-focus layer $o_{0}$ (SIO output);
•
$i_{j}$ is the SIO j-th pre-processed image;
•
$M_{j}(o_{0},o_{d})$ is the imaging model, which will be further described below;
•
σ
j2 is the noise variance, which we assume to be homogeneous here;
•
λ
 is the regularization parameter and is set to 0.3 as explained in [22];
•
$S_{o_{0}}$ and $S_{o_{d}}$ are the power spectral densities of each object layer;
•
$\tilde{.}$ refers to the 2D discrete Fourier transform of its argument and *f* is the 2D spatial frequency.

The noise variances σ
j2 and the object power spectral densities $S_{o_{0}}$ and $S_{o_{d}}$ , are estimated from the data in an unsupervised way [22,27].

The imaging model corresponding to the j-th image reads:

(2) $$\begin{array}{ll} M_{j}(o_{0},o_{d}) & =[h_{0}⋆(m_{j,0}.t_{j}[o_{0}]){]}_{\text{III}}(k,l)\\ & +[h_{d}⋆(m_{j,d}.t_{j}[o_{d}]){]}_{\text{III}}(k,l) \end{array}$$

where ⋆
 depicts the discrete 2D convolution product and $t_{j}[.]$ is a subpixel shift operator that computes the shifted discretized object. ${\left[.\right]}_{\text{III}}$ is a downsampling operator that makes it explicit that the observed object $\left(o_{0},o_{d}\right)$ can be oversampled with respect to the SIM images $i_{j}$ to ensure that the reconstructed super-resolved image satisfies the Shannon-Nyquist sampling theorem. The imaging model depends on 5 parameters: the in-focus and defocused point-spread functions (PSF) $h_{0}$ and $h_{d}$ , the in-focus and defocused illumination patterns $m_{j,0}$ and $m_{j,d}$ and the retinal shifts, which were estimated after the image selection process. These retinal shifts determine the subpixel shift operator $t_{j}[.]$ . The choice or estimation of these parameters, required for the reconstruction process, is described in Appendix A.

It should be noted that no image registration is performed on the SIO frames during the SIO reconstruction process. Instead, the estimated retinal shifts are taken into account in the BOSSA-SIM algorithm. Finally, we note that the count of selected SIO frames at the end of the image selection stage differs from one image sequence to another. This discrepancy arises because microsaccades, blinks and AO quality degradation occur at disparate times and frequencies across the image sequences. The number of SIO frames per orientation of the pattern processed for each image sequence is as follows: 79 for subject A, 70 for subject B and 50 for subject C.

## 3. Results

**Fig. 5.**
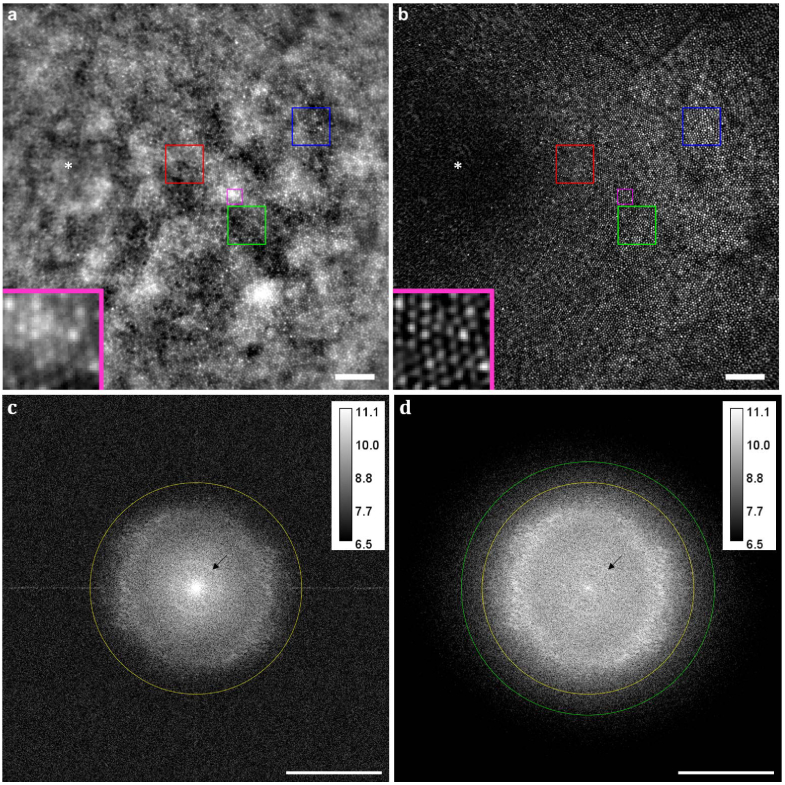
Conventional wide-field versus SIO $2.54^{\circ }\times 2.54^{\circ }$ retinal images, computed from the same raw data acquired on subject A. a-d, Conventional wide-field image (a) and SIO image (b) together with their corresponding image spectra (c) and (d) respectively. Insets (a-b), magnified views of the magenta boxed $0.1^{\circ }\times 0.1^{\circ }$ region. Scale bars, 0.25^∘^ (a-b), 100 cycles/degree (c-d). The displayed images are focused on the photoreceptor layer and centered at 0.9^∘^ eccentricity. To correct for the uneven background illumination that falls off toward the edge of the field of view, a bandpass filter was applied to the wide-field image (see Appendix B). In a-b, the white asterisk marks the location of the foveal center. A linear lookup table (LUT) with a saturation of the 0.3% of the brightest and darkest pixels is applied to a-b. In c-d, the yellow (resp. green) circle indicates the effective cutoff frequency of the wide-field image (resp. SIO image), above which the spectrum is dominated by the noise. The image spectra are displayed in logarithmic scale. The blue, green and red boxed regions in a-b are magnified on Fig. 7.

In order to investigate the potential of SIO for super-resolved retinal imaging, we imaged the cone photoreceptors at about 1^∘^ nasal, in three healthy subjects. This section highlights the results obtained on subject A, while SIO reconstructions acquired on subjects B and C are shown and analyzed in Appendix E. To compare SIO with conventional flood-illumination ophthalmoscopy under the same imaging conditions (light flux, AO correction, eye movement), a wide-field image was constructed from the SIO frames used for the SIO reconstruction. The computation of the wide-field images is performed as follows. Firstly, the SIO frames are registered by shifting them by the opposite of the retinal shifts estimated during the SIO reconstruction process. Then, the temporal average of the registered SIO frames is computed. We checked that the illumination patterns are effectively removed by this averaging and registration process so as to produce a reliable conventional flood-illumination image. Hereafter, the flood-illumination image will be referred to as the wide-field image. The comparison between the wide-field and SIO images is shown in Fig. 5. In the wide-field image (Fig. 5(a)), even after correction of the uneven background illumination (see Appendix B), Fig. 10), the contrast of the cone mosaic is dominated by the out-of-focus background, which comes from defocused layers and choroidal scattering [28]. In the SIO image (Fig. 5(b)), this defocused contribution is rejected by optical sectioning and the cones are more visible. The optical sectioning effect is also noticeable in the image spectra (Fig. 5(c)-d): the bright central peak corresponding to low spatial frequency energy in the wide-field image spectrum is greatly reduced in the SIO image spectrum. Furthermore, the ring outlined by the black arrow in Fig. 5(c), which is characteristic of the retinal pigment epithelium (RPE), a layer just beneath the photoreceptor layer, is easily seen in the wide-field image spectrum whereas it is barely visible in the SIO image spectrum. These observations show how the optical sectioning of SIO greatly reduces the contributions of scattering light background and out-of-focus layers and consequently improves the contrast of the in-focus retinal layer.

**Fig. 6.**
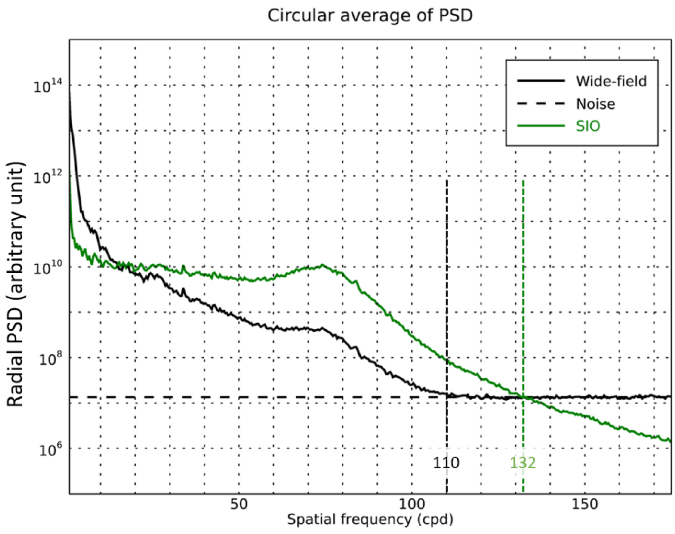
Estimation of the effective cutoff frequency for the wide-field and SIO images on subject A. The plotted curves correspond to the circular average of the power spectral densities of the wide-field image (solid black line), the noise (dashed black line) and the SIO image (solid green line).

**Fig. 7.**
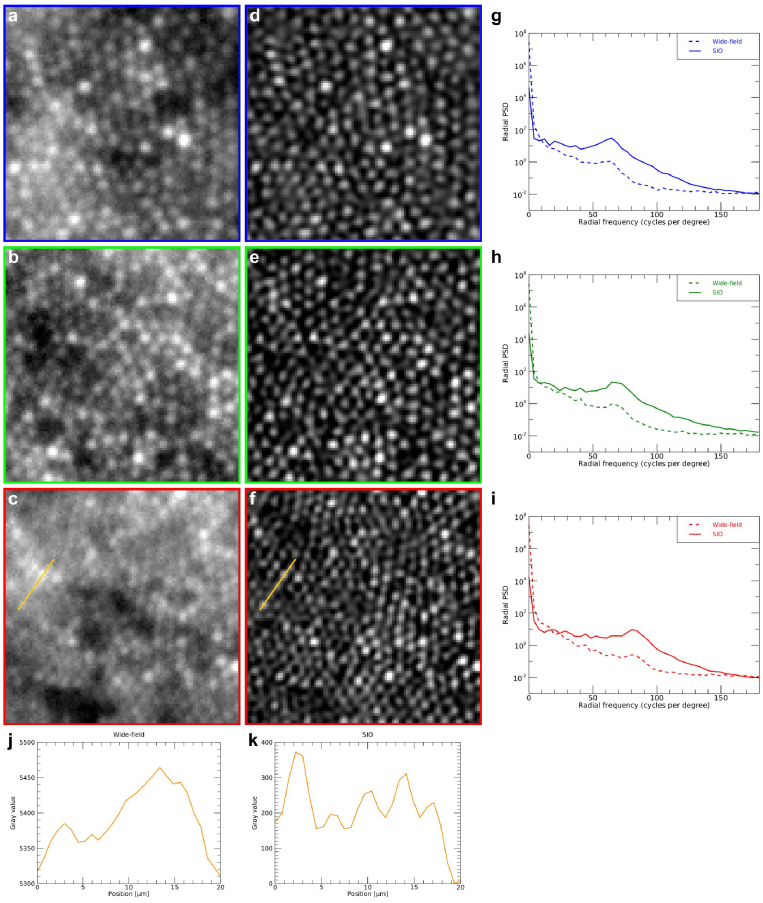
Comparisons of the wide-field and SIO retinal images over $0.25^{\circ }\times 0.25^{\circ }$ enlarged views. a-c, Magnification of the blue (a), green (b) and red (c) boxed regions in the wide-field image (Fig. 5(a)), d-f, Corresponding magnification from the SIO image (Fig. 5(b)), g-i, Radial power spectra of the blue (g), green (h), respectively red (i) boxed regions, j-k, Plots of the image intensity along the 20 *μ *m lines drawn in c (j) and f (k). The blue, green and red boxed regions are centered at retinal eccentricities of 1.6^∘ ^, 1.2^∘ ^, and 0.7^∘^ respectively. A linear LUT with a saturation of the 0.3% of the brightest and darkest pixels is applied to the images displayed in a-f.

In the wide-field image spectrum, the diffraction-limited cutoff frequency is not reached because of the noise. We thus defined the wide-field effective cutoff frequency as the radial frequency above which the wide-field image power spectral density (PSD) estimated as the image periodogram, goes down to the noise PSD. The latter is estimated using the unsupervised method proposed in BOSSA-SIM [22]. As shown in Fig. 6, the evaluation of the effective cutoff frequency yields $f_{c,\text{eff}}^{WF}=110$ cycles/degree for the wide-field image. This value is symbolized by the yellow circle in Fig. 5(c)-d. Similarly, in the SIO image, the effective cutoff frequency is given by the radial frequency above which the SIO image PSD goes down to the noise PSD. This yields an effective SIO cutoff frequency $f_{c,\text{eff}}^{SIO}$ of 132, representing a 20% increase over the conventional wide-field effective cutoff frequency. Hence, SIO achieves resolution improvement with respect to the effective resolution of the conventional wide-field image as well as optical sectioning. Fig. 7 provides further comparisons between the wide-field and the SIO images over enlarged views of $0.25^{\circ }\times 0.25^{\circ }$ field-of-view. The radial power spectra plotted in Fig. 7(g)-i, exhibit a peak at 65 cycles/degree (g), 69 cycles/degree (h) and 81 cycles/degree (i) related to the photoreceptor average density in each of the $0.25^{\circ }\times 0.25^{\circ }$ regions of interest. These peaks reach higher values for the SIO, which shows that the cone mosaic has a higher contrast in the SIO image. At $0.7^{\circ }$ eccentricity (Fig. 7(c) and (f)), the SIO is able to resolve cones that are indistinguishable in the wide-field image, as exemplified in the intensity plots (Fig. 7(j)-k). In particular, the spacing of the last two cones resolved only in the SIO intensity plot is 2.7μ
m corresponding to a spatial frequency of 111 cycles/degree, which is slightly higher than the wide-field effective cutoff frequency.

The comparisons of conventional wide-field and SIO images acquired on subjects B and C, provided in Appendix E, concur with the above assessment: SIO achieves higher resolution and contrast than conventional wide-field ophthalmoscopy.

## 4. Discussion

**Fig. 8.**
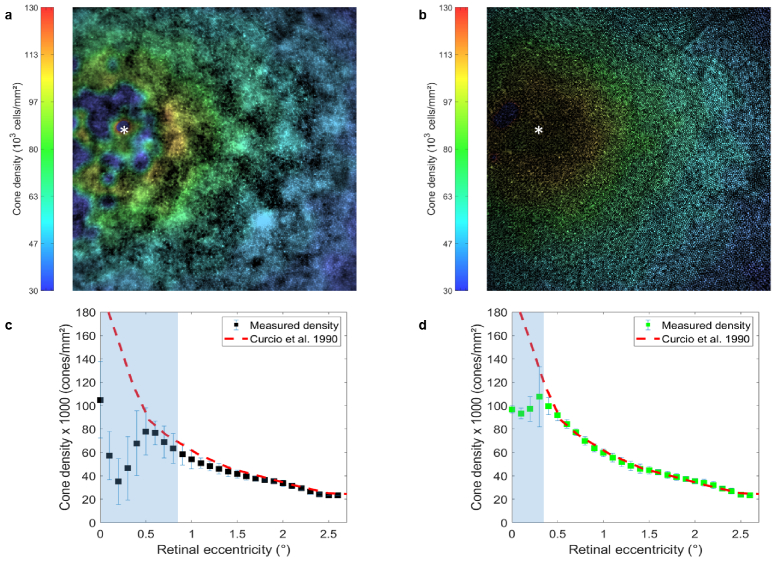
Measurement of the cone density. (a-b) Cone density distribution color maps obtained from the wide-field image (a) and from the SIO image (b) acquired on subject A. (c-d) The estimated cone density mean (squares) and standard deviation (blue lines) values as a function of the retinal eccentricity respectively computed from a and b. The white asterisk in a,b, indicates the foveal center. The red dashed line corresponds to histological measurement from an average retina [31]. The blue shaded region in c,d, indicates the eccentricities for which the cone density estimated from each image is unreliable (ie., when the ratio of standard deviation over the mean value is greater than 0.1 ).

The higher resolution and contrast achieved by SIO compared with conventional wide-field ophthalmoscopy makes SIO a highly valuable tool for imaging foveal cones and monitoring cone density, which is a biomarker commonly used for detecting the early onset of common retinal degenerative diseases [29][30]. To investigate this matter, we compared the cone density map computed from the SIO reconstructed image with the one computed from the conventional wide-field image, as shown in Fig. 8. The computation of the cone density maps is detailed in Appendix C.

For both images, the measurements are consistent with histology up to a retinal eccentricity below which the estimated densities start to deviate as the cones are no longer resolved. We defined the retinal eccentricity below which the measurements are unreliable as that for which the ratio of the standard deviation over the mean value of the estimated cone density becomes greater than 10% (blue shaded region in Fig. 8(c-d)). Using this criterion, it appears that the relative number of cones that can be identified is improved in the SIO image compared to the conventional wide-field image. Moreover, unlike SLO, SIO images are distortion-free, which is of the utmost importance for longitudinal studies of cone density.

**Fig. 9.**
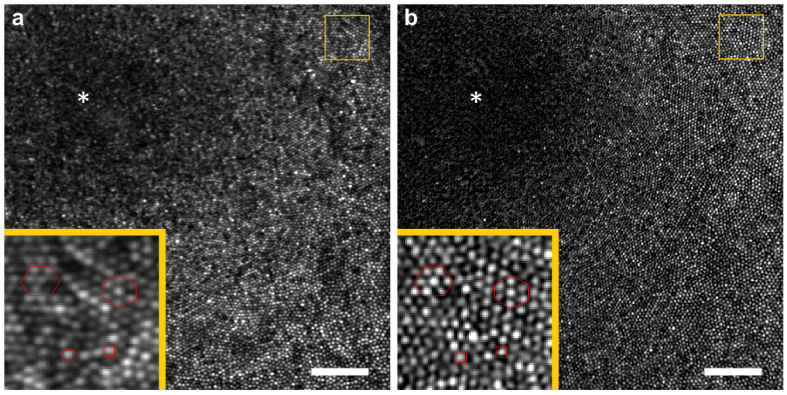
cSLO versus SIO $1.74^{\circ }\times 1.74^{\circ }$ retinal images. (a) cSLO image, (b) SIO image. Scale bars, 0.25^∘ ^. The insets display a magnification of the yellow boxed $0.2^{\circ }\times 0.2^{\circ }$ regions. In boths insets, the red hexagone and square shapes highlighting common cones were drawn to facilitate the image comparison. The white asterisk indicates the foveal center. The data were acquired on subject A. A linear LUT with a saturation of the 0.3% of the brightest and darkest pixels was applied to both images.

**Fig. 10.**
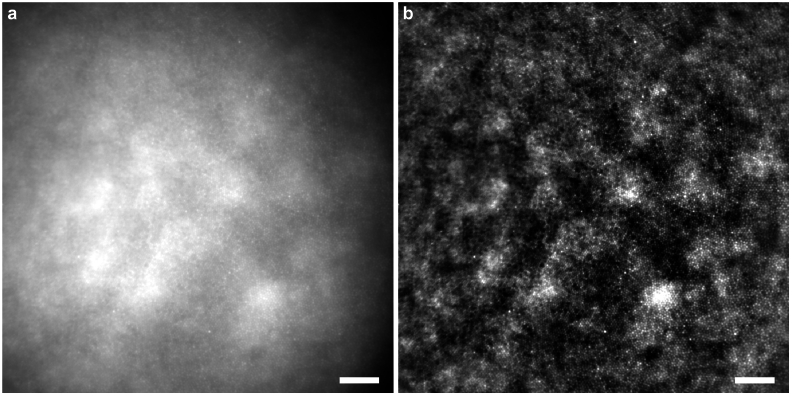
Correction of the heterogeneous background in the conventional wide-field image. (a) $2.54^{\circ }\times 2.54^{\circ }$ wide-field image, (b) Wide-field image after applying a highpass filter. Scale bars, 0.25^∘ ^. A linear LUT with a saturation of the 0.3% of the brightest and darkest pixels was applied to both images.

A comparison between the SIO image and the image acquired with a commercial AO-corrected cSLO (MAORI, Physical Sciences Inc., Andover MA USA) is presented in Fig. 9. The MAORI system has an imaging wavelength of 780nm and its pinhole diameter was set to 1 Airy unit during the acquisition. More information about the commercial AO-cSLO that we used is provided in Appendix D. We observe that the cones appear more contrasted and sharper in the SIO image. It is worth mentioning that the cone brightness is not the same from one imaging modality to the other due to the temporal variability of photoreceptor reflectance [32,33]. Furthermore, SIO and the MAORI cSLO qualitatively achieve a similar optical sectioning. This result was expected, as it was already demonstrated in microscopy that SIM and confocal imaging enable similar axial sectioning [20]. Our SIM implementation to flood-illumination ophthalmoscope narrows the gap between wide-field systems and confocal scanning laser systems, particularly in terms of optical sectioning and image contrast. In both SIM and sub-Airy cSLO, an asymptotical twofold maximum gain in optical cutoff frequency can theoretically be obtained: for SIM it corresponds to a modulation frequency at the optical cutoff frequency (for which the fringe contrast tends towards 0), for the sub-Airy cSLO it corresponds to an infinitely small detection pinhole. However, the use of SIM for super-resolution *in vivo* retinal imaging is currently hindered by the signal-to-noise ratio (SNR) in the data, which limits the achievable resolution gain provided by SIM. In contrast, super-resolution retinal imaging has already been demonstrated in recent research grade AO cSLO [34][35] using sub-Airy detection.

For SIO, similarly to SIM, the resolution enhancement depends on the ratio between the fringe contrast and noise. The low contrast of the fringe patterns in the raw SIO frames (see Fig. 3) is due to two reasons. The first reason is that the fringe pattern is projected onto the retina, thus the contrast of the fringes is attenuated by the optical transfer function (OTF) of the instrument at the modulation spatial frequency of the fringes, twice (once during fringe projection, once during imaging). Even if there is an AO correction that compensates for the ocular aberrations, there are still residual aberrations that degrade the OTF compared to an ideal diffraction-limited case. The second reason is that the fringe contrast is reduced by a strong background, which mostly comes from the multiple scattering of the light propagating inside the eye [36,37]. This background contribution to the wide-field image also decreases the SNR. Due to a poor SNR, the diffraction limit is not reached and the effective resolution is limited by the noise in the acquired frames.

A possible development to reduce the multiple scattering contribution to the raw SIO frames would be to combine SIO with partial-field illumination [37] or rolling slit ophthalmoscopy [38]. This would allow one to further exploit the resolution improvement enabled by SIO and incidentally to reduce the number of required frames for SIO reconstruction as the SNR in the raw frames would be higher.

As a final note, although we designed our SIO for a reflective AO-corrected flood-illumination imaging modality, it is worth mentioning that fundus autofluorescence imaging could also benefit from the enhanced resolution enabled by SIM. A SIM implementation without AO for fundus autofluorescence imaging of the RPE in the living retina has been proposed in [19]. We anticipate that combining adaptive optics with structured illumination could enable high-resolution imaging of RPE cells. Furthermore, the diffuse background, which compromises contrast in wide-field retinal images, can be spectrally filtered out in autofluorescence RPE imaging, thereby enhancing the SNR in the resulting SIO raw frames.

## 5. Conclusion

In conclusion, we have used an AO-corrected flood-illumination ophthalmoscope and demonstrated *in vivo* structured illumination ophthalmoscopy (SIO) on the human retina. We have shown that structured illumination brings optical sectioning to wide-field ophthalmoscopy while improving its resolution. The SIO exploits the BOSSA-SIM reconstruction algorithm, which presents five main features that make it successful in reconstructing high resolution contrasted retinal images. Firstly, it is based on an imaging model that takes into account the retinal shifts. Secondly, its original 2-layer object model, which distinguishes the in-focus signal from the out-of-focus contribution, enables one to jointly achieve optical sectioning and super-resolution from two-dimensional data [22]. Thirdly, thanks to the Bayesian framework, the method is robust to noise, which can be important in wide-field retinal images. Fourthly, its reconstruction hyper-parameters (object and noise power spectral densities) are adjusted in an unsupervised fashion (*i.e.*, automatically) from the data. Lastly, the method imposes a positivity constraint on the reconstructed object, which is known to induce spectral extrapolation for objects on a dark background. Thus, this positivity constraint, combined with the optical sectioning, contributes to an extension of the reconstruction frequency bandwidth in addition to the resolution improvement brought by SIM.

By imaging the cone mosaic near the fovea on three subjects, we have shown contrast and resolution enhancement in the SIO reconstructed images compared with the corresponding conventional wide-field images. This paves the way towards accurate photoreceptor mapping in patients with a small pupil. More generally, AO-corrected structured illumination provides a generic strategy to achieve super-resolution and optical sectioning in a wide variety of biomedical imaging applications, despite sample motion, spurious scattering and aberrations.

## Figures and Tables

**Fig. 11.**
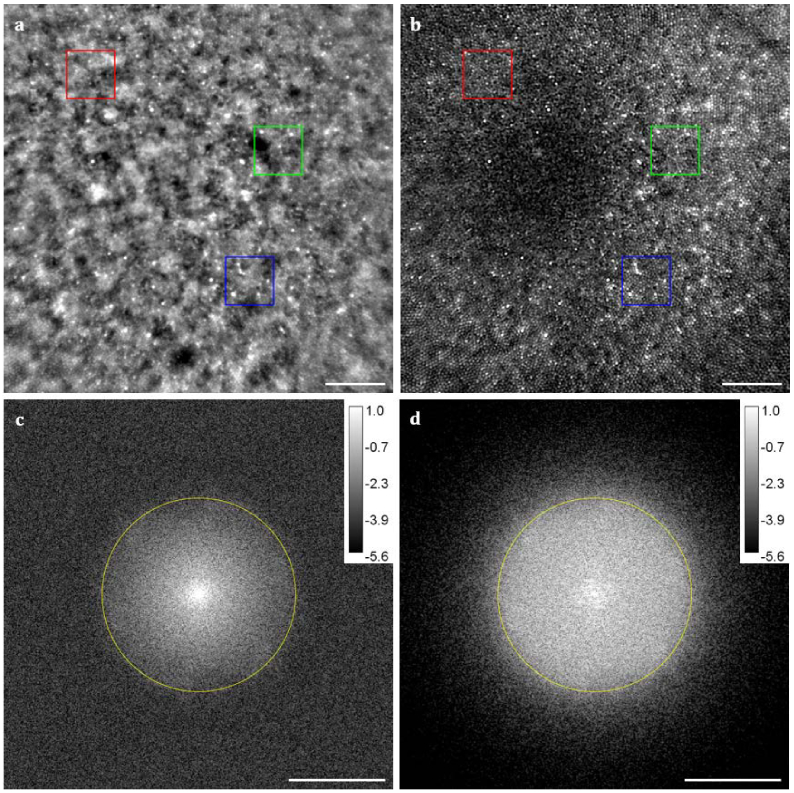
Conventional wide-field versus SIO $1.65^{\circ }\times 1.65^{\circ }$ retinal images computed from the same raw data, subject B. Conventional wide-field image (a) and SIO image (b) together with their corresponding image spectra (c) and (d) respectively. Insets (a-b), magnified views of the magenta boxed $0.1^{\circ }\times 0.1^{\circ }$ region. Scale bars, 0.25^∘^ (a-b), 100 cycles/degree (c-d). The displayed images are focused on the photoreceptor layer near the fovea. To correct for the uneven background illumination that falls off toward the edge of the field of view, a bandpass filter was applied to the wide-field image (see Appendix B). A linear lookup table (LUT) with a saturation of the 0.3% of the brightest and darkest pixels is applied to a-b. In c-d, the yellow circle indicates the effective cutoff frequency of the wide-field image (100 cycles/degree), above which the wide-field image spectrum is dominated by the noise. The image spectra are displayed in logarithmic scale. The blue, green and red boxed regions in a-b are magnified on Fig. 12.

**Fig. 12.**
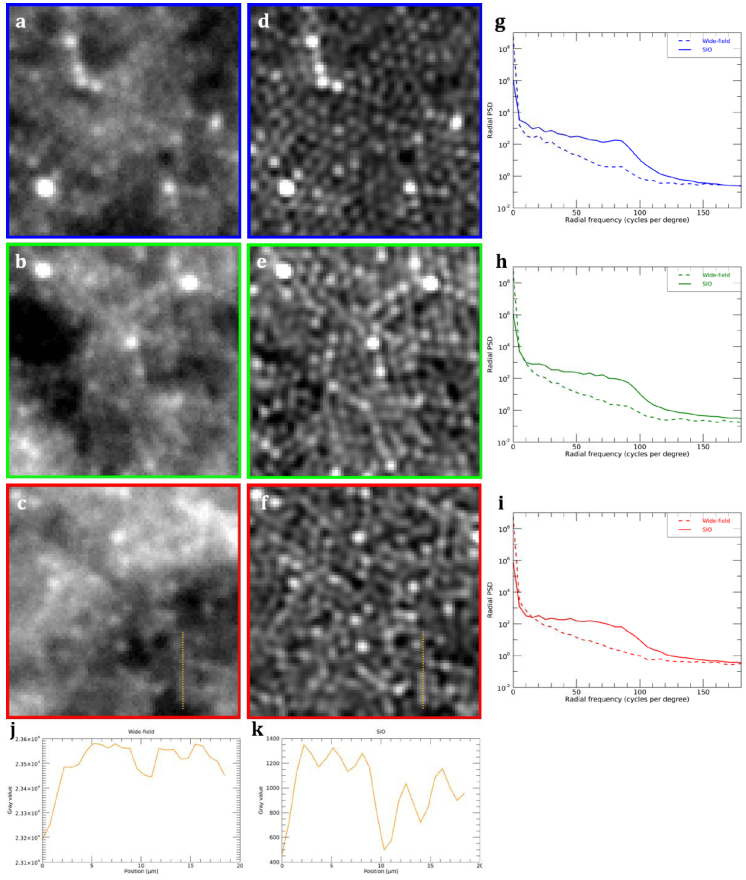
Comparisons of the wide-field and SIO retinal images over $0.2^{\circ }\times 0.2^{\circ }$ enlarged views, subject B. (a-c) Magnification of the blue (a), green (b) and red (c) boxed regions in the wide-field image (Fig. 11(a)), d-f, Corresponding magnification from the SIO image (Fig. 11(b)), (g-i) Radial power spectra of the blue (g), green (h), respectively red (i) boxed regions, j-k, Plots of the image intensity along the 18 *μ *m lines drawn in c (j) and f (k). A linear LUT with a saturation of the 0.3% of the brightest and darkest pixels is applied to the images displayed in (a-f).

**Fig. 13.**
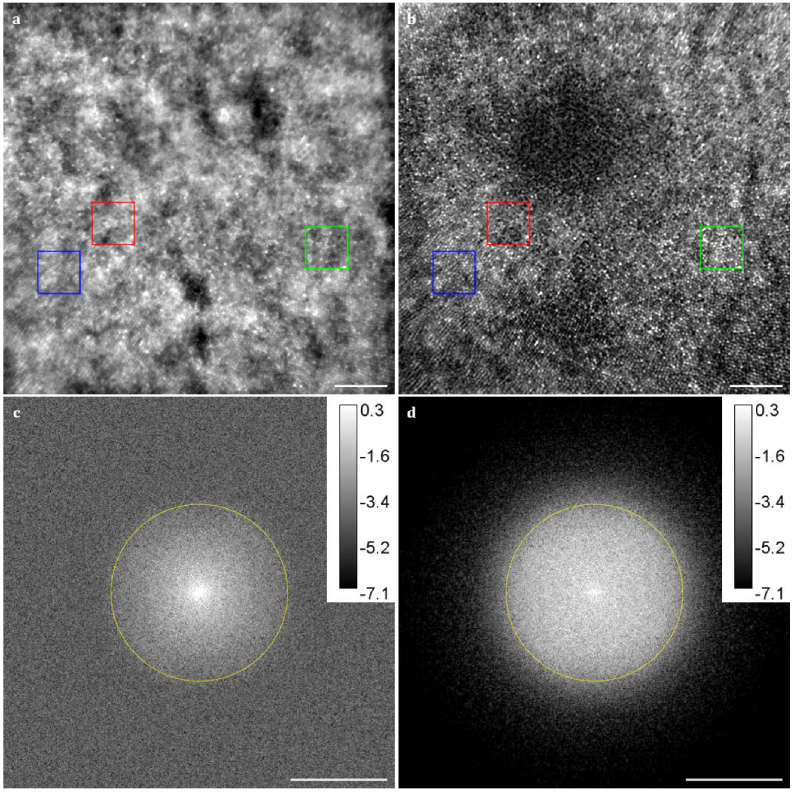
Conventional wide-field versus SIO $1.84^{\circ }\times 1.84^{\circ }$ retinal images computed from the same raw data, subject C. Conventional wide-field image (a) and SIO image (b) together with their corresponding image spectra (c) and (d) respectively. Insets (a-b), magnified views of the magenta boxed $0.1^{\circ }\times 0.1^{\circ }$ region. Scale bars, 0.25^∘^ (a-b), 100 cycles/degree (c-d). The displayed images are focused on the photoreceptor layer near the fovea. To correct for the uneven background illumination that falls off toward the edge of the field of view, a bandpass filter was applied to the wide-field image (see Appendix B). A linear lookup table (LUT) with a saturation of the 0.3% of the brightest and darkest pixels is applied to (a-b). In (c-d), the yellow circle indicates the effective cutoff frequency of the wide-field image (91 cycles/degree), above which the wide-field image spectrum is dominated by the noise. The image spectra are displayed in logarithmic scale. The blue, green and red boxed regions in (a-b) are magnified on Fig. 14.

**Fig. 14.**
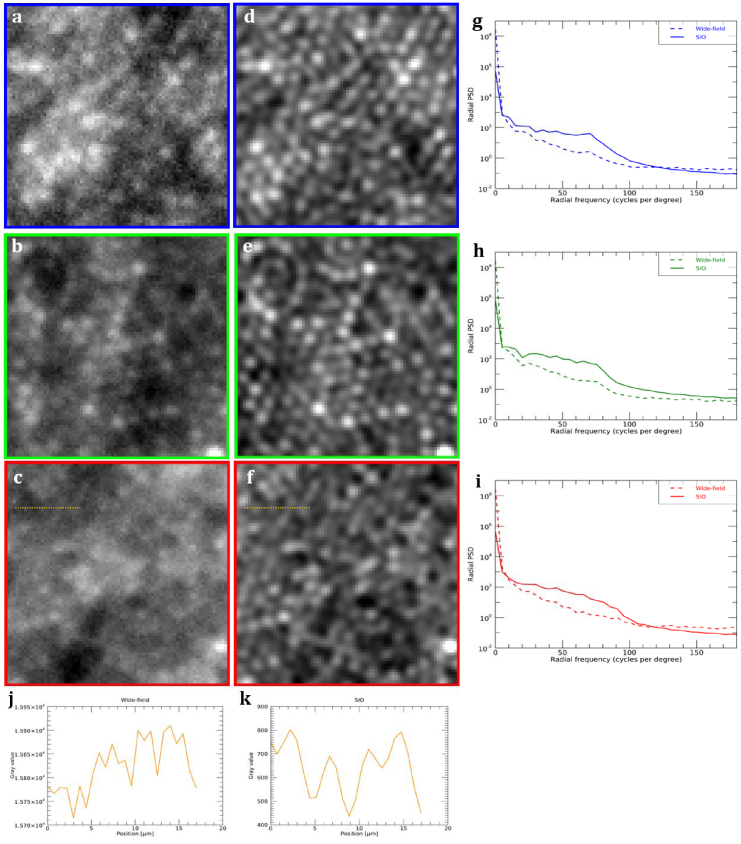
Comparisons of the wide-field and SIO retinal images over $0.2^{\circ }\times 0.2^{\circ }$ enlarged views, subject C. (a-c) Magnification of the blue (a), green (b) and red (c) boxed regions in the wide-field image (Fig. 13(a)), (d-f) Corresponding magnification from the SIO image (Fig. 13(b)), (g-i) Radial power spectra of the blue (g), green (h), respectively red (i) boxed regions, j-k, Plots of the image intensity along the 17 *μ *m lines drawn in c (j) and f (k). A linear LUT with a saturation of the 0.3% of the brightest and darkest pixels is applied to the images displayed in (a-f).

## Data Availability

Data underlying the results presented in this paper are not publicly available at this time but may be obtained from the authors upon reasonable request.

## Appendices

### A. Choice of the parameters for the SIO reconstruction

The parameters’ choice of the imaging model (Equation (2)), required for the SIO reconstruction, is described below.

The PSFs are derived from the corresponding optical transfer functions (OTF) in the reciprocal space. The in-focus OTF ${\tilde{h}}_{0}$ is modeled by a diffraction-limited OTF attenuated by an exponential term with a damping parameter of 0.1 as in FairSIM [39] to account for residual optical aberrations. The out-of-focus OTF ${\tilde{h}}_{d}$ is obtained by applying the same exponential attenuation to an OTF with defocus aberration. The defocus value was chosen in such a way that the defocused OTF value at the modulation spatial frequency $f_{m}$ is null.

As we project only two sinusoidal patterns, one oriented at θ
1=−
45∘
 and the other at θ
2=+45∘
 , there are two sets of patterns $\{m_{\theta ,0},m_{\theta ,d}{\}}_{\theta }$ to be estimated (See Equation (2)). The in-focus illumination pattern at a given orientation *θ *, is given by:

(A1)
mθ
,0(r→
)=Iθ
(r→
)[1+α
θ
(r→
)cos⁡
(2π
f→
θ
,m.r→
+ϕ
θ
)]

with $\vec{r}=(k,l)$ the 2D spatial coordinates. The intensity map $I_{\theta }$ takes into account the heterogeneous background due to inhomogeneities in the illumination beam and to multiple scattering effects inside the eye (see Fig. 10). $I_{\theta }$ was estimated by fitting a third-order polynomial on the average SIO frame for the given *θ *. To account for variable modulation contrasts over the field of view, a contrast map α
θ
 of values between 0.1 and 0.3 was set. Finally, the spatial frequency ${\vec{f}}_{\theta ,m}$ and the phase ϕ
θ
 of the illumination pattern were estimated using a least square fit of a sinusoidal fringe model over a filtered version of the average SIO frame for the given *θ *. The purpose of the filtering is to filter out the object structures that may skew the pattern parameter fitting.

As we incoherently project a fringe pattern onto the retina, the fringe contrast decreases with defocus and thus, the illumination patterns are only contrasted at the in-focus object plane. Hence, we can consider that the defocused illumination pattern $m_{\theta ,d}$ is not modulated:

(A2)
$$m_{\theta ,d}(\vec{r})=I_{\theta }(\vec{r})$$

### B. Correction of the heterogeneous background in the conventional wide-field images

Due to the illumination inhomogeneities and mostly to the scattering of the light when propagating inside the eye [36,40], retinal wide-field images contain a strong background which slightly decreases toward the edges of the field [37]. As shown in Fig. 10(a), this background significantly reduces the contrast of the retinal structures that are imaged (in our case, the foveal cones). As the background is composed of low spatial frequencies, we can filter it out by applying a highpass filter to the wide-field image. The filtering of the wide-field image was performed using ImageJ’s "bandpass filter" function. The parameters were set to filter large structures down to 100 pixels and small structures up to 0 pixel. The resulting filtered image is displayed in Fig. 10(b). One drawback of this background correction is that it also suppresses the low spatial frequency of the observed retinal layer.

### C. Computation of the cone density map

To generate a pointwise density map, we divided the cone mosaic image into an overlapping grid of 64×
64 pixel (corresponding to 88 μ
m×
88 μ
m ) regions of interest (ROIs), where each ROI was displaced from the previous one by 10 pixels (corresponding to 15μ
m ). These values were chosen empirically to provide a good trade-off between pointwise accuracy and map smoothness. Then, cone density was computed for each ROI using a fully automated algorithm based on modal spacing as described in [41]. Bicubic image interpolation was then used to increase the size of the cone density map in order to match the cone mosaic image.

### D. Acquisition of the cSLO retinal images

In order to compare SIO with a confocal scanning laser ophthalmoscope, additional retinal images were acquired with a commercial AO-corrected cSLO system (MAORI, Physical Sciences Inc., Andover MA USA) [42]. Image acquisition was performed on the same subject as for the SIO and in the same conditions, *i.e.*, with neither pupil dilation nor cyclopegia, in a dark room. The optical power entering the eye was set to 800μ
W at 780 nm for the illumination source and 700μ
W for the wavefront sensor source at 840 nm. 100 cSLO $2^{\circ }\times 2^{\circ }$ individual frames centered on the fovea were acquired at 24 Hz. They were then processed using the MAORI image processing library for registration and distortion compensation [43]. Figure 9(a) shows the processed cSLO image, which was manually registered with the SIO image.

### E. Additional SIO reconstructions

Comparisons of AO-corrected conventional wide-field and SIO reconstructions acquired on subjects B and C at around $1^{\circ }$ nasal are presented here. The wide-field images were computed from the SIO frames used for the SIO reconstruction in order to compare both imaging modalities under the same imaging conditions (light flux, AO correction, eye movement). The computation of the wide-field images is performed as follows. Firstly, the SIO frames are registered by shifting them by the opposite of the retinal shifts estimated during the SIO reconstruction process. Then, the temporal average of the registered SIO frames is computed. Figure 11 and Fig. 12 (respectively Fig. 13 and Fig. 14) compare the SIO reconstruction and the wide-field image acquired on subject B (respectively subject C).

Both SIO reconstructions present a cone mosaic, which exhibits better contrast compared with the wide-field images. The darker area of circular shape in both SIO reconstructions corresponds to the foveal pit, located at the center of the fovea. As the imaging system were focused on the photoreceptors near $1^{\circ }$ nasal during the acquisitions, the foveal pit is out-of-focus and appears dark in the SIO image due to the optical sectioning. Furthermore, we can observe that in the image spectrum of both SIO reconstructions (Fig. 11(d) and Fig. 13(d)), the frequency bandwidth is extended with respect to the effective cutoff frequency symbolized by the yellow circle as it was shown for the SIO reconstruction on subject A (see Fig. 5).

The intensity profile plotted in Fig. 12 shows that SIO is able to resolve a cone mosaic of period 2.9 *μ *m, which corresponds to a spatial frequency of 102 cpd, a value slightly above the effective cutoff frequency of the corresponding wide-field image (100 cpd). This is a proof of the resolution improvement with respect to the effective cutoff frequency enabled by SIO. The intensity profile plotted in Fig. 14 underlines how the optical sectioning achieved by SIO contributes to a better resolving capability. Indeed, the wide-field image has a strong background, which dominates the photoreceptor’s signal in the area around the profile line. In the SIO reconstruction, this background is filtered out through optical sectioning and we are able to distinguish four cones along the intensity profile. In the wide-field image, the intensity profile is noisy and it is hard to distinguish the intensity variations caused by the presence of a cone from noise.

We can note that the resolution improvement achieved by SIO is less pronounced in images from subjects B and C compared to those from subject A. This discrepancy is mainly due to larger AO performance fluctuations during the acquisition on subject B and C, comparatively to subject A. This increased variability affects the modulation contrast in processed SIO frames for subjects B and C, leading to a decreased resolution improvement. Additionally, the number of frames processed for SIO reconstruction differs across subjects: 79 frames per orientation for A, 70 for B and 50 for C. Consequently, the lower frame count for subjects B and C results in a reduced SNR in both the processed wide-field and SIO images.
